# Electrochemical and optical biosensors for early-stage cancer diagnosis by using graphene and graphene oxide

**DOI:** 10.1186/s12645-017-0035-z

**Published:** 2017-12-11

**Authors:** Aditya Balaji, Jin Zhang

**Affiliations:** 1 Department of Biomedical Engineering, University of Western Ontario, 1151 Richmond St., London, ON N6A 5B9 Canada; 2 Department of Chemical and Biochemical Engineering, University of Western Ontario, 1151 Richmond St., London, ON N6A 5B9 Canada

**Keywords:** Graphene, Graphene oxide, Protein–protein interaction (PPI), Electrochemical sensor, Optical sensor

## Abstract

Conventional instruments for cancer diagnosis including magnetic resonance imaging, computed tomography scan, are expensive and require long-waiting time, whilst the outcomes have not approached to the successful early-stage diagnosis yet. Due to the special properties of graphene-based nanocomposites, e.g., good electrical and thermal conductivity, luminescence, and mechanic flexibility, these ultra-thin two-dimensional nanostructures have been extensively used as platforms for detecting biomolecules and cells. Herein, we discuss the development of two types of graphene and graphene oxide-based biosensors: electrochemical and optical, aimed for tumor detection and early diagnosis of cancer. Moreover, we highlight the challenges of their use as biosensors for cancer detection. Efficient surface modification and suitable bio-conjugation of graphene and graphene oxide is discussed, including key role in improvement of the biocompatibility, and improved performance in terms of selectivity and sensitivity towards the early diagnosis of cancer.

## Background

According to World Health Organization (WHO), over 8.8 million people worldwide died from cancer in 2015, and it represents the first main cause of death in the United States (Forouzanfar et al. 2016). Cancer is caused by the uncontrolled growth and spread of abnormal cells. Cancer cells can evade apoptosis as these malignant tumor cells keep dividing and undergo different stages (Herr 2011). Firstly, there is proto-oncogenesis that initiates the cell division and mutation of these genes that generate cancer-related genes. Secondly, mutated tumor suppressor genes lead to the cancer formation. Thirdly, mutations of gene regulated on apoptosis tend to be carcinogenic. Lastly, mutations of the DNA repairing genes also increase the chances of leading to cancer. These mutations that occur may arise from deletion, duplication, or insertion of the nucleotides (Ecsedy and Hunter 2008). General cancer treatment techniques are normally associated with delineating the cancer cells at the early stages like chemotherapy, surgery, and radiation. Therefore, diagnosis of cancer is vital for timely individuating an effective cancer treatment. However, traditional diagnostic tools, including magnetic resonance imaging (MRI), computed tomography (CT), and X-ray scan, are expensive and normally require a long-waiting time to access fullstop. Furthermore, traditional diagnostic tools require several million cells for accurate clinical diagnosis, which is far to reach the success for any early diagnostics of cancer (Hsieh et al. 2016). The challenges of early diagnosis and effective treatment of cancer requires a sensitive sensor to detect small amount of samples with high sensitivity and selectivity. For instance, an ideal molecular imaging is expected to correctly diagnose early-stage tumor of approximately 100–1000 cells (Ecsedy and Hunter 2008).

Nanoparticles with average particles size in the range from 1 to 100 nm have been considered as an alternative tool for cancer diagnosis and therapy at early stage as they have special physiochemical size-dependent properties (SalmanOgli 2011; Chinen et al. 2015). Quite recently, graphene and graphene oxide, the ultra-thin two-dimensional nanomaterials, have attracted extensive attention because of their unique structure and remarkable mechanical, electrical, thermal, and optical properties. More studies have shown that nanoparticles incorporating with graphene or graphene oxide could show great potentials for sensing cancer biomarkers or cells at very low concentration, and realizing targeted treatment (Feng et al. 2013; Ma et al. 2013).

In this review, recent key findings of the hybrid graphene-based biosensor for detecting cancer cells are summarized. Surface modification of hybrid graphene/graphene oxide used in electrochemical biosensors and optical biosensors for detecting cancer biomarkers or cancer cells is addressed.

## Graphene and graphene OXIDE and surface modifications

Graphene is an allotrope of carbon which forms a 2D, atomic scale hexagonal lattice and it is able to effectively conduct heat and electricity. The electron configuration of carbon is1*s* ^2^2*s* ^2^2*p* ^2^. In the excited state, four equivalent quantum–mechanical states are formed through the *sp* ^*n*^ hybridisation, which plays an essential role in four covalent carbon bonds. The stability of graphene is dependent on how tightly packed carbon atoms are from the *sp* ^2^ hybridization. Graphene is a zero-gap semiconductor due to the conduction and valence band meet at the Dirac points (Nair et al. 2008). Fascinating properties of graphene arise from its high surface area combined with electronic and thermal conductivity and its mechanical strength. Due to the material’s high surface area-to-volume ratio and high conductivity, it leads to significant improvements in many applications (Zurutuza et al. 2014).

Due to the unique microstructures, graphene demonstrates special and always enhanced physiochemical properties. For instance, the Young’s modulus and the intrinsic strength of graphene are around 1 TPa and 130 GPa, respectively. The room temperature electron mobility of graphene is 2.5 × 10^5^ cm^2^ V^−1^ s^−1^. The thermal conductivity of the graphene is above 3000 W mK^−1^ (Nair et al. 2008). Graphene has been used to fabricate flexible electronics (Gorbachev et al. 2012), high-frequency transistors, and logic transistor (Britnell et al. 2012). In addition, single-layer and double-layer graphene products normally show optical transparency of 98% (Li et al. 2008a). With suitable design, graphene layers can become transparent simultaneously at high drive voltage. Recent studies show that graphene can be applied in electrochemical and optical biosensors to detect small amounts of cancer biomarkers (Ma et al. 2013).

**Fig. 1:**
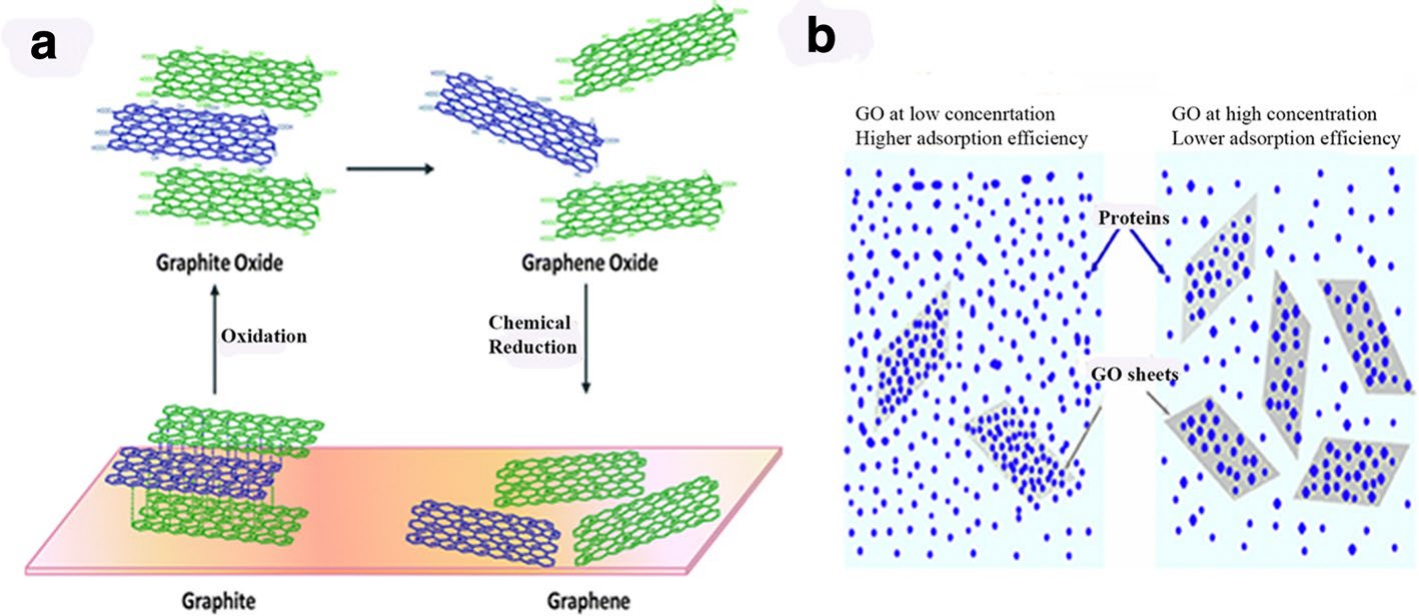
**a** The chemical process of graphene oxide and Graphene. **b** Schematic illustration where a low graphene oxide (GO) concentration exhibits a high adsorption of proteins in the left depiction. The right depiction illustrates a high GO concentration with low adsorption of proteins (The copyright permission is obtained from Wei et al. (2015))

Hummer’s approach is the most common one for the synthesis of graphene sheets from graphene oxide as shown in Fig. 1 (Yin et al. 2015; Li et al. 2008b; Chen et al. 2013; Toh et al. 2014; Zhu et al. 2010). The reduced form of graphene oxide is graphene through an oxidizing agent, like hydrazine or ascorbic acid.

The oxygen functional groups on the surface of graphene oxide (GO) provide good sites for myriad of interactions for linking molecules such as polymers, nanoparticles (NPs). In GO, the associated functional groups are epoxy bridges, hydroxyl, and carboxyl groups. Due to the disruption of *sp* ^2^ bonding, it acts as an electrical insulator. The advantages of using GO for enzyme immobilization induce many explorations of its properties and applications where techniques, such as atomic force microscopy (AFM) and scanning electron microscopy (SEM), could be used to view the immobilized structure (Yin et al. 2015; Li et al. 2008b; Chen et al. 2013; Toh et al. 2014; Zhu et al. 2010). Different enzymes have been used to modify the surface of graphene oxide. The adsorption efficiency is dependent on the material concentration and the protein concentration as the correlation (1)

1 $$\text{AE}= W_{\text{ad}} /W_{\text{g}},$$

where AE represents the adsorption efficiency, *W*_ad_ represents the qualitative measure of the adsorbed protein, and *W*_g_ represents the qualitative measure of GO (Wei et al. 2015).

The relationship between the material and the protein complex is shown in Fig. 1b. The synthesis of graphene sheets can be done using the Hummer’s approach (Yin et al. 2015; Li et al. 2008b; Chen et al. 2013; Toh et al. 2014; Zhu et al. 2010). Graphene Oxide (GO) is produced from graphite oxide and can be derived from one of the famous processes via the Hummer’s Method as shown in Fig. 1. The reduced form of graphene oxide is graphene through an oxidizing agent, like hydrazine or ascorbic acid. Overall, using the Hummer’s approach achieves the yield of both compounds.

Graphene and graphene oxide with suitable surface modifications have been applied in the bio-conjugation studies aimed for cancer cells detection. Table 1 lists major recognizing elements modified on graphene-based nanomaterials and targeted molecules related to cancer cells.

**Table 1:** Target molecule and common recognizing elements modified on graphene/graphene oxide for detecting cancer cells

Targeted molecules	Surface modification and recognizing elements	Disease	References
DNA-t	DNA-C and DNA-r.AuNP	Breast cancer	Rasheed et al. (2014)
Nucleolin	Aptamers, e.g., AS1411	Any type of cancer	Feng et al. (2011)
Anti-CRP antibody	CRP antibody	Lung cancer, colorectal cancer, myeloma cancer, prostate cancer	Zhu (2015)
PCG	PPi	Melanoma cancer	Muthuraj et al. (2017)
Folate receptor	Folate-modified hydrophilic polymers	Epithelial-derived tumors	Li et al. (2016)
Anti-CRP antibody	HRP anti-CRP antibody	Lymphoma cancer	Zhu et al. (2010)
Ab-DNA	Au nanoparticle-tagged DNA	Breast cancer cells	Jung et al. (2010)

## Graphene-based electrochemical biosensor for cancer cell detection

### Electrochemical mechanism

The electrochemical design consists of a three-electrode system: the working electrode (WE), the counter electrode (CE), and the reference electrode (RE). The electrolyte plays a vital role in having a chemical substance conjugating and doping onto the specific material. The cyclic voltammetry (CV) is where the voltage gets tested between the reference electrode and the working electrode Another electrochemical technique involved is the electrochemical impedance spectroscopy (EIS) where the applications range from studying the corrosion of metals, adsorption, and desorption to electrode surface, electrochemical synthesis of materials, catalytic reaction kinetics, and ions mobility in energy storage devices such as batteries and supercapacitors (Chang and Park 2010). EIS technology can distinguish the electrochemical behavior between the coating and the metal substrate through the use of built in electrical circuits such as resistors and capacitors (Gan et al. 2015; Chang and Park 2010; Orazem et al. 2011).

### Graphene-based biosensors for detecting cancer cells by using electrochemistry

Breast Cancer 1 (BRCA 1) gene is a tumor suppressor gene that is expressed in breast cells and other tissues. Mutations associated with this gene lead to high risk of breast cancer. This technology implemented can detect the concentration level of BRCA 1 levels within the human body. Breast Cancer 2 (BRCA 2) gene is another tumor suppressor gene that is expressed. Mutations associated with BRCA 1 and BRCA 2 resulted in 54 and 23% for ovarian cancer, respectively (King et al. 2003; Futreal et al. 1994).

**Fig. 2:**
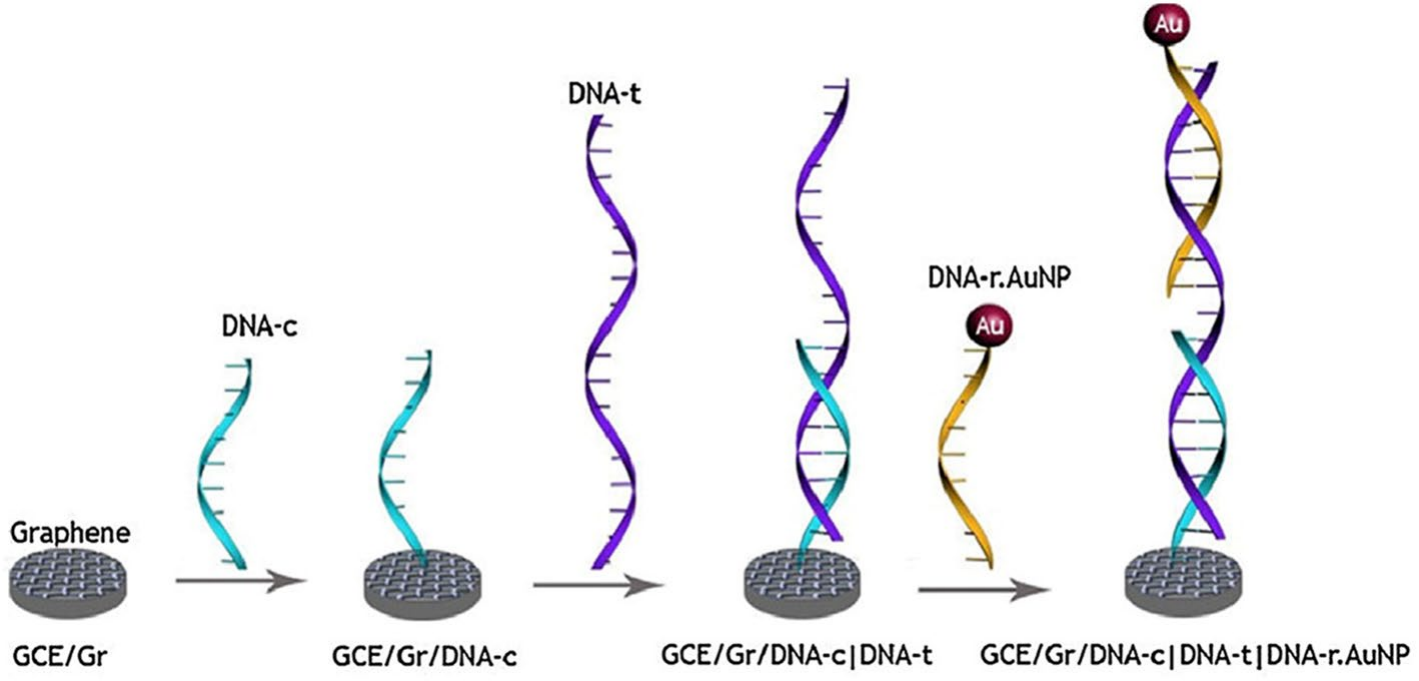
CV results from different concentrations of DNA-t (Copyright permission is obtained from Rasheed et al. (2014))

Figure 2 indicates the electrochemical sensor for detecting BRCA 1 gene, the target DNA (DNA-t). First graphene-decorated glass carbon electrode (GCE) is modified with single-strand DNA sequence (DNA-c) to hybridize the target DNA (DNA-t). The hybridization of probe DNA (DNA-r) modified on Au nanoparticles (NPs) will occur on the surface of graphene-decorated GCE. The amount of Au NP conjugated on the graphene modified electrode can result in the increase of the oxidation peaks in CV measurement (Rasheed et al. 2014). This designed electrochemical sensor could detect 1 fM BRCA1 gene which is the biomarker of breast cancer.

Another design on the three-electrode cell is established where the working/sensing electrodes consist of modified MDEAs, Ag/AgCl as the reference electrode, and BSA/anti-CRP antibodies/MPA/rGO-NP/ITO on the counter electrode. Eight circular electrodes are immersed onto one Indium Titanium Oxide electrode (Feng et al. 2011).

**Fig. 3:**
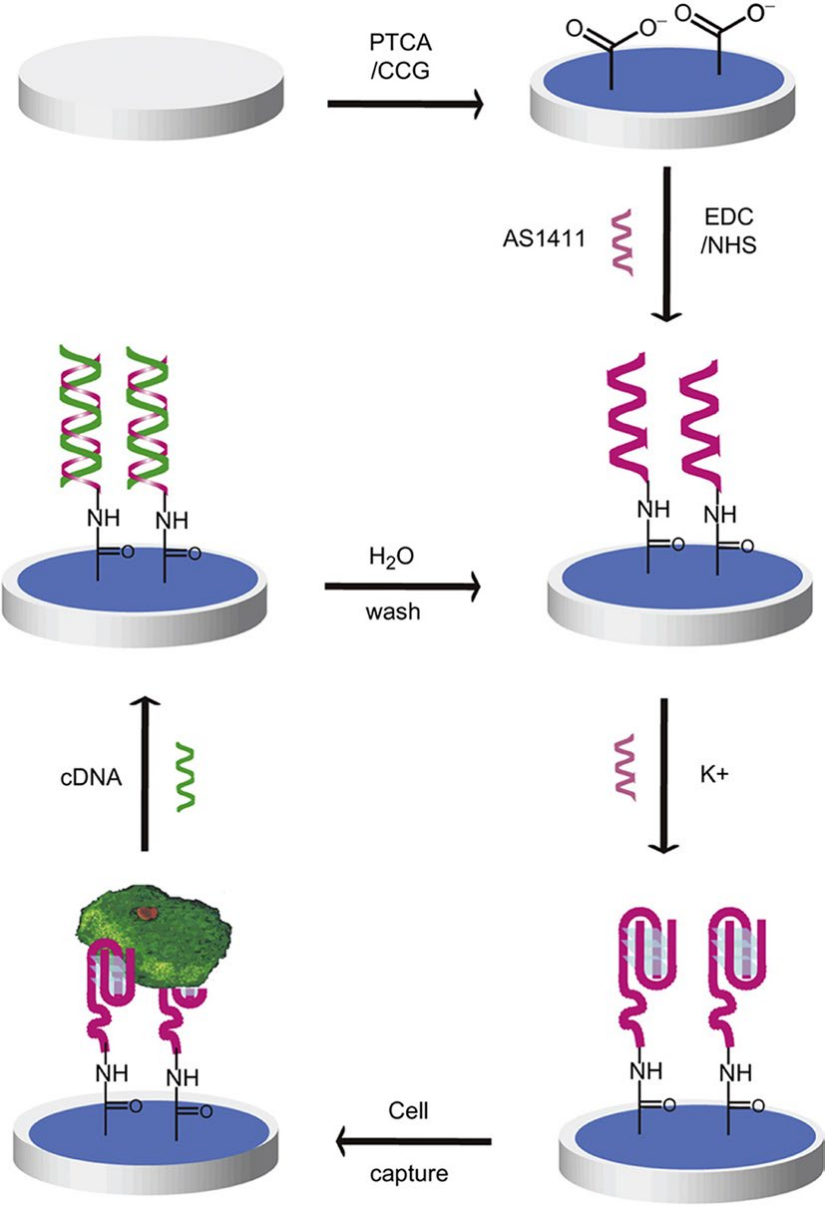
Schematic illusion of the biosensor made by aptamer/DNA depositing on graphene (Copyright permission is obtained from Feng et al. (2011))

Using the chronoamperometry analysis, the redox current had a linear proportion with increasing DNA-t concentration. When 10 fM of DNA-t was deposited, it showed a high current value as opposed to 3 mM DNA-t. This system proved that the sensor is selective towards DNA and can be utilized for detecting mismatches in BRCA 1 gene mutations as shown in Fig. 3 (Rasheed et al. 2014).

The detection capability of the GCE/Gr/DNA-c|DNA-t|DNA-r.AuNP with different DNA-t concentrations was monitored at 1.1 V. With an increase in concentration of DNA-t, the change was detectable up to 100 aM DNA-t (Rasheed et al. 2014).

**Fig. 4:**
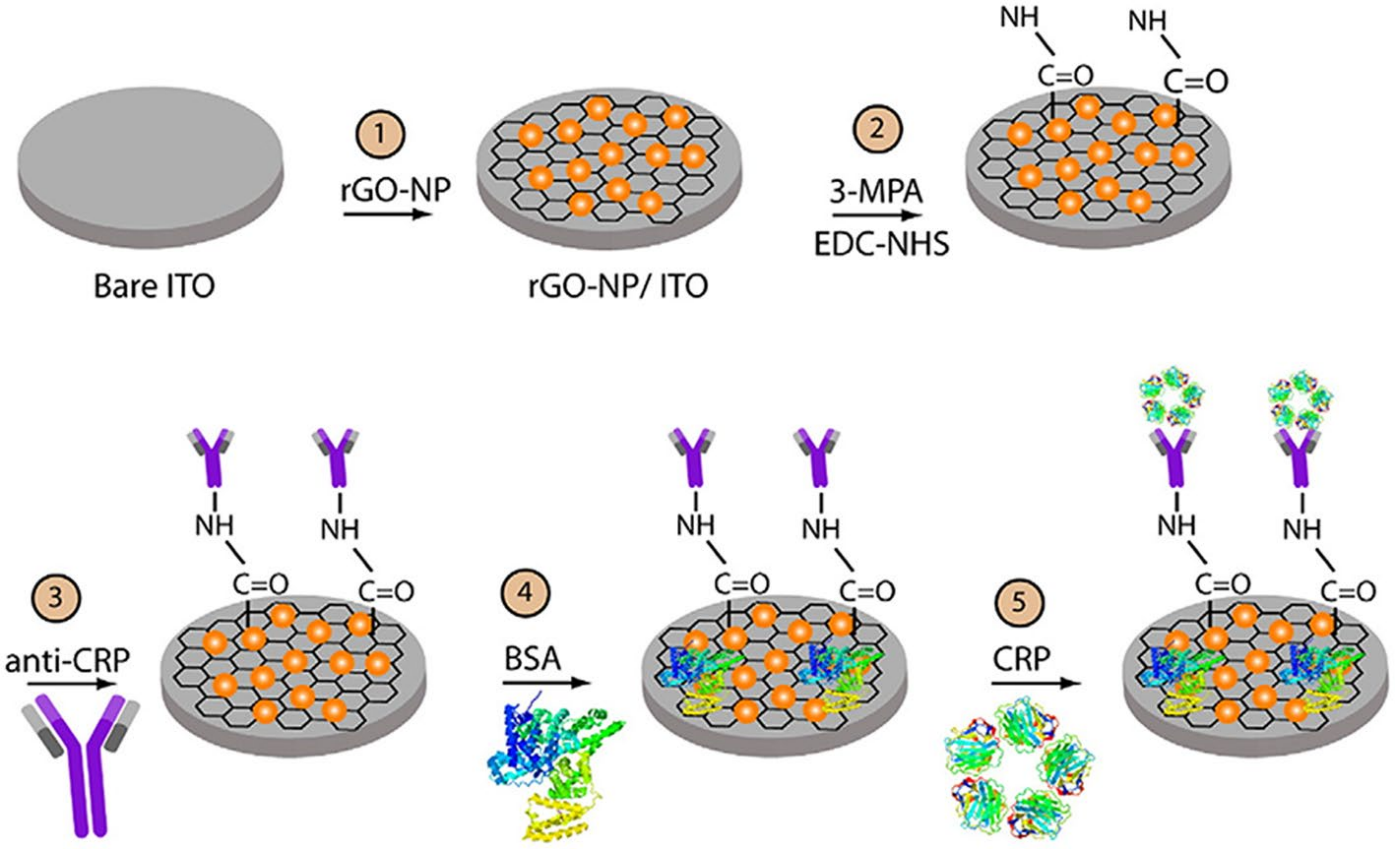
Schematic illustration of the graphene oxide with surface modification used as an electrode in a three-electrode cell (Copyright permission is obtained from Yagati et al. (2016))

The EIS technique helps in detecting the electrode surface composition by monitoring the electron transfer resistance (*R*_et_). From different compositions involved where Nafion was used to immobilize the nanocomposite layer, a lower *R*_et_ value was achieved compared to the involvement of using an aptamer. With higher impedance value, cancer cell detection based on aptamer-graphene-modified electrode is suited the best as shown in Fig. 4. The technique implemented provides the high binding affinity of AS 1411 to the nucleolin surface of cancer cells. The EIS assists in indicating the affinity level of the aptamer to the cancer cells compared to normal cells (Feng et al. 2011).

HeLa cells, K 562 cells, MDA 231 cells, and NIH 3T3 cells were tested in this work. The cells were measured using EIS and found that the breast cancer cell line, MDA-231, had the highest *R*_et_ value. From this, the conclusion is that the MDA-231 cells monitored the cancer cells much better than the other type of cells using the cell culture technique (Feng et al. 2011).

The EIS technique is also essential for the impedance detection of C-Reactive Protein (CRP). Different concentrations of the CRP were coated onto the electrode with respect to anti-CRP antibodies. The increase in binding affinity of CRP with anti-CRP instigated the increase in *R*_ct_. With a higher impedance value, it can be driven that the surface-modified species is immobilized onto the electrode as shown in Fig. 4 (Yagati et al. 2016).

The detection capability of the GCE/Gr/DNA-c|DNA-t|DNA-r.AuNP with different DNA-t concentrations was monitored at 1.1 V. With an increase in concentration of DNA-t, the change was detectable up to 100aM DNA-t (Rasheed et al. 2014).

## Graphene-based optical biosensors for cancer cell detection

### Mechanism of optical biosensors

For optical sensors, different types of mechanisms involving energy transfers are involved such as Forster Resonance Energy Transfer, Fluorescence Energy Transfer, Resonance Energy Transfer, or Electronic Energy Transfer (Chang and Park 2010). Theodor Forster developed this technique where the mechanism influences energy transfers between two light-sensitive molecules. The energy transfer mechanisms involve when a donor chromophore is in its high excitation state and energy is transferred to the acceptor chromophore through dipole–dipole interactions (Chang and Park 2010; Orazem et al. 2011). The criteria for the stated energy mechanisms involve the distance between the donor and acceptor chromophore, and the orientation of the chromophores. The methods used in these energy transfers help in identifying how surface modification will take place. The FRET mechanism is between two fluorophores used as donor (D) and acceptor (A). The energy transfer efficiency (*E*, i.e., the fraction of energy transferred) is reverse proportional to the distance of two fluorophores as shown in Eq. 2.

2 $$E = \frac{1}{{\left[ {1 + \left( {\frac{r}{{R_{o} }}} \right)^{6} } \right]}},$$

where *r* is the distance between two fluorophores and *R*_0_ the distance at which 50% *E* was achieved. *R*_0_ is a characteristic parameter for given partners at given medium.

Chemiluminescence Resonance Energy Transfer (CRET) is another technique used when non-radiative energy source is used to transfer energy from the donor chromophore to the acceptor chromophore. Chemiluminescence (CL) is one of the techniques implemented to generate electromagnetic radiation by which the excited product initiates back to its original state before the excitation. Using the CRET methods, immunosensors can be established in receptiveness to the C-reactive protein (CRP) levels. CRP measure levels can differentiate between the normal and serious conditions. (Gupta et al. 2014; Masters 2014; Beljonne et al. 2009; Zhu 2015; Huang et al. 2006). This is one of the other unique techniques implemented on the surface modification on graphene oxide or graphene.

The other technique implemented to explain the use of the optical sensors is the photothermal therapy, often referred to as PTT. It uses electromagnetic radiation within the infrared region to treat medical conditions, such as the elimination of the tumor cells. Using PTT has better advantages than using PDT as oxygen is not involved to interact with the target cells or tissues and lower energy is used, thus minimizing the cytotoxicity of the cells. Photothermal therapy has been a novel technique in eliminating the defects of chemotherapy (Chen et al. 2016).

### Design of graphene-based optical probe for cancer cell detection

Here, we are focusing on two major optical biosensors, i.e., Fluorescence Resonance Energy Transfer (FRET), Chemiluminescence Energy Resonance Transfer (CRET).

FRET and CRET are non-radiation fluorescence. The distance-dependent energy resonance transfer between donor and acceptor makes them offer great benefits in accurately detecting biomolecules/cells in vivo and in vitro.

Fluorescent amino acid (histidine)-functionalized perylenediimide (PDI-HIS) is a technique where the “turn-off and turn-on” can detect Cu^2+^ ions. The disaggregation of PDI-HIS-Cu^2+^ of the fluorescence quenching helps detect the PPi levels (Muthuraj et al. 2015).

A unique optical approach on detecting the concentration of pyrophosphate (PPi) has a direct correlation with the cancer diagnosis. The fabrication technique of using the fluorescent probe of PDI-HIS, copper ion, and graphene oxide (GO) which enhances the selectivity and sensitivity for detecting PPi, a cancer biomarker (Muthuraj et al. 2015). The results show that the self-assembled nanocomposites made of PCG (PDI-His + GO + Cu^2+^) have a low detection limit (LOD), 1fM, for PPi in comparison to PDI-HIS-Cu^2+^.

The B16F10 cells were used with GO-based composites to detect the concentration of PPi. After incubation time, the B16F10 cells treated with 300 μg mL^−1^ of PDI-HIS give a red fluorescence. The addition of Cu^2+^ and GO will quench the fluorescence. PCG is much more sensitive in detecting the level of PPi than only PDI-HIS + Cu^2+^ (Muthuraj et al. 2015).

**Fig. 5:**
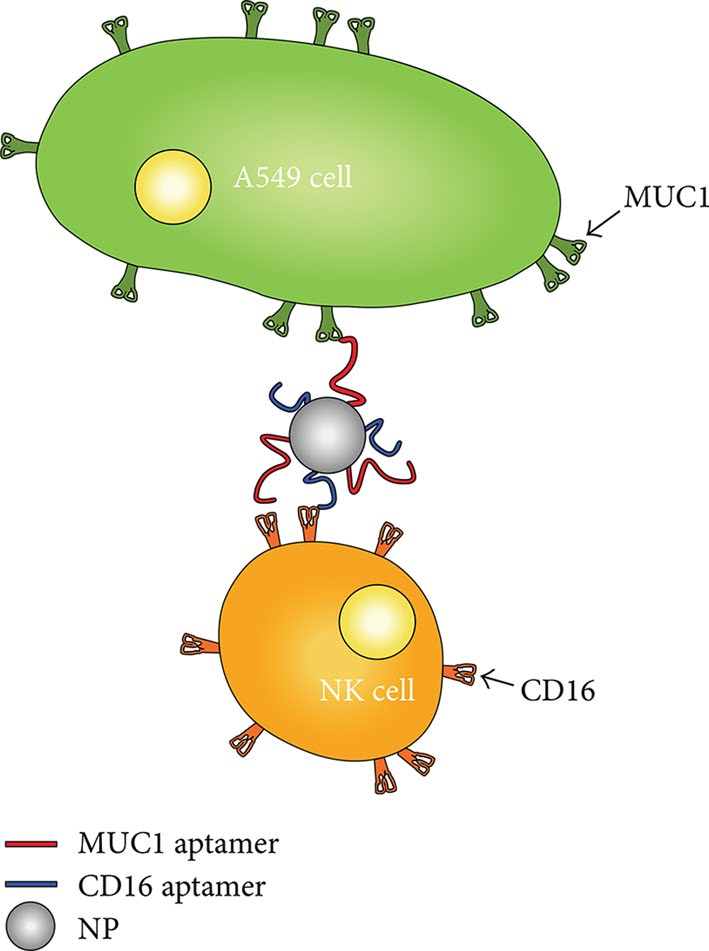
An amphipathic nanoparticle is attached to MUC1 and CD16 aptamers. These aptamers attach to the MUC-1 positive tumor cells and CD16 positive tumor cells that can help bring the NK cell to the vicinity of the MUC-1 positive cancer cell which acts as an anticancer cell (Copyright permission is obtained from Yu et al. (2015))

The overexpression of MUC1 has been noted in cancer cells with regard to features associated biochemically and functionally (Papadimitriou et al. 1999). MUC1 consists of MUC1-N and MUC1-C regions where MUC1-N is composed of proline, threonine, and serine-rich domain. In the mitochondria and nucleus, there is a detection of MUC1-C in the mitochondria and nucleus, whereas MUC1-N is detected in the nuclear speckles as shown in Fig. 5. Looking at a comparison between a normal epithelial cell and a cancer cell, one can distinguish that the tumor cell has lost its polarity where the and the increased expression of the hypoglycosylated form of MUC1 (Yu et al. 2015; Yang et al. 2013; Nath and Mukherjee 2014; Pouilly et al. 2000; Beatson et al. 2010; Mukherjee et al. 2003; Bitler et al. 2009; Sahraei et al. 2012). MUC1 is present on the surface of the tumor cells, close to the growth factors surrounding it (Chen et al. 2015). Antichemotherapy drugs are inaccessible to the targets as the glycosylated MUC1 inhibits from reaching its targets. MUC-1 has a correlation with the increase in tumor cells related to breast, ovarian, colon, lung, and prostatic cancers (Chen et al. 2015). The new novel immunotherapy instigated that the increase in MUC-1 would have to diminish by attaching it to the NK cells which makes this an anticancer method (Yu et al. 2015; Yang et al. 2013; Nath and Mukherjee 2014; Pouilly et al. 2000; Beatson et al. 2010; Mukherjee et al. 2003; Bitler et al. 2009; Sahraei et al. 2012).

**Fig. 6:**
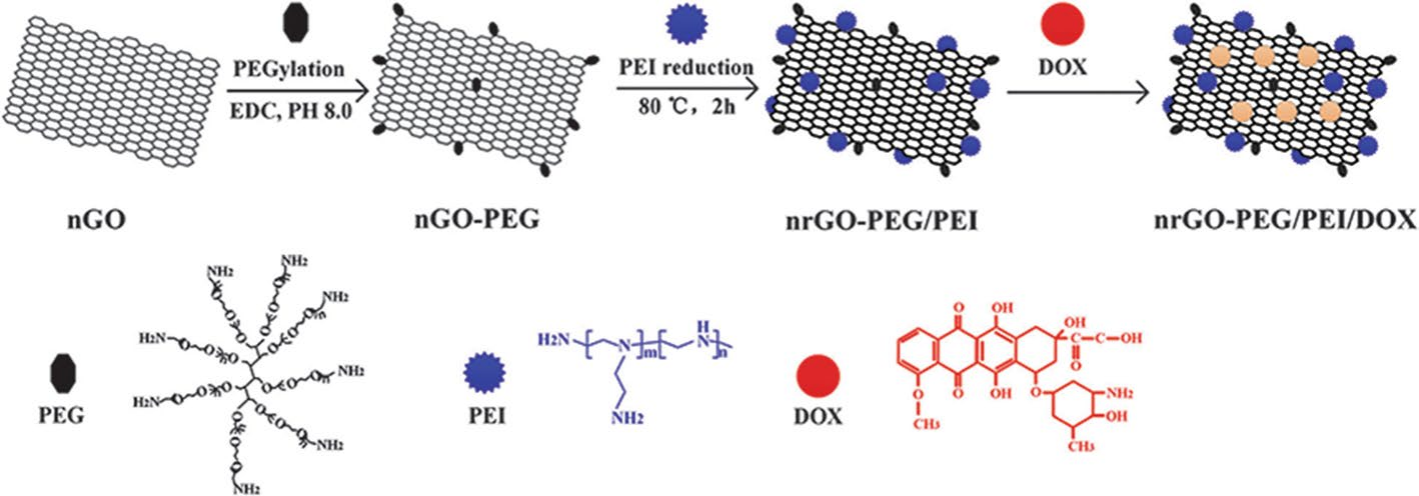
Schematic reaction of the synthesized nrGO-PEG/PEI/DOX (Copyright permission is obtained from Li et al. (2016))

In addition, FRET technique by utilizing quantum dots for the chemotherapy of ovarian cancer has been reported. The FRET technique transfers energy to the drug molecule from the quantum dot (QD) as they are attached on graphene. The fluorescence emission was recorded and the quenching indicates the release of doxorubicin (DOX) from QD. A more innovative modified structure shown in Fig. 6 exhibits high anticancer efficiency by conjugating DOX on to the QD modified with a glycoprotein, e.g., MUC 1 which realizes the targeted delivery (Savla et al. 2011). Some reports show that graphene or graphene oxide-based FRET sensor incorporating the design with antibody-DNA-Au NP can be used for detecting cancer cells (Jung et al. 2010).

CRET techniques apply luminescence organic chemicals to excite an acceptor in CRET pair. The graphene-based CRET sensor has been developed for detecting the interaction between anti-C-Reactive Protein (CRP) and the C-Reactive Protein (CRP). Such immune sensor can accurately detect the C-reactive protein level. The amount of CRP with respect to the normal levels is usually less than 3 mg L^−1^. The concentration of CRP significantly increases when there is an infection associated with cardiovascular disease, in this case, the primary issue is focused towards Lymphoma Cancer. Higher CRP concentrations have been reported towards lung, pancreatic, breast, ovary, esophagus, liver, biliary tract, stomach, and multiple myeloma (Heikkila et al. 2007).

The new stepping stone of surface-modified Apt-PBMC and the CRP-capturing ability is examined (Hwang et al. 2016). The new innovation which drives as a stepping stone fluorescence imaging towards the detection of CRP has been examined. The new surface-modified engineering application is a new innovative idea towards cancer treatment. The Apt-PBMC complex had a recognition towards different concentrations of CRP and had an impact towards the fluorescence intensity levels. As the concentrations of the CRP increased, the fluorescence intensity increased (Hwang et al. 2016).

## Conclusions and perspectives

This paper reviews the recent development of graphene and graphene oxide used for detecting cancer cells. Graphene oxide/graphene can be used as the transducer towards the application in cancer treatment because of their superior properties in terms of electronic/thermal conductivity, and special luminescence and mechanical properties. Two major biosensor systems addressed here include electrochemical and optical Sensors. The electrochemical technique delves into concepts with CV, EIS, and amperometry, the cells involved for the design to study the properties of the surface modification. The fluorescence and chemiluminescence biosensors produce different properties when exposed to the surface-modified graphene oxide/graphene. In general, graphene/graphene oxide with suitable surface modification can detect the specific interactions between the recognizing elements and target molecules for certain cancer cells. Though numerous studies on the surface modification and the different capping concentration levels have been performed, it is still not clear what are the optimal conditions for the best detection and target for cancer cells. In addition, the in vivo behavior of graphene oxide and graphene with surface modification is still under investigation. The major challenges outlined with these nanomaterials: (i) biocompatibility in vivo distribution, (ii) chemical modifications for sensing applications and cell membrane penetration, (iii) understanding of in vitro and in vivo toxicity profiles. In conclusion, with the challenges addressed properly to produce the optimal design, the graphene-based nanomaterials will significantly benefit to the early diagnosis of cancer cells.
